# “We don’t trust all data coming from all facilities”: factors influencing the quality of care network data quality in Ethiopia

**DOI:** 10.1080/16549716.2023.2279856

**Published:** 2023-11-29

**Authors:** Asebe Amenu Tufa, Geremew Gonfa, Anene Tesfa, Theodros Getachew, Desalegn Bekele, Ftalew Dagnaw, Nehla Djellouli, Tim Colbourn, Tanya Marchant, Seblewengel Lemma

**Affiliations:** a Ethiopian Public Health Institute, HSRHRD, Addis Ababa, Ethiopia; b Ethiopian Ministry of Health, Quality and Clinical Service Directorate, Addis Ababa, Ethiopia; c Institute for Global Health, University College London, London, UK; d Department of Disease Control, London School of Hygiene & Tropical Medicine, London, UK

**Keywords:** Quality of care network, data quality, data reliability, data learning, data sharing

## Abstract

**Background:**

Good quality data are a key to quality health care. In 2017, WHO has launched the Quality of Care Network (QCN) to reduce maternal, newborn and stillbirth mortality via learning and sharing networks. Guided by the principle of equity and dignity, the network members agreed to implement the programme in 2017–2021.

**Objective:**

This paper seeks to explore how QCN has contributed to improving data quality and to identify factors influencing quality of data in Ethiopia.

**Methods:**

We conducted a qualitative study in selected QCN facilities in Ethiopia using key informant interview and observation methods. We interviewed 40 people at national, sub-national and facility levels. Non-participant observations were carried out in four purposively selected health facilities; we accessed monthly reports from 41 QCN learning facilities. A codebook was prepared following a deductive and inductive analytical approach, coded using Nvivo 12 and thematically analysed.

**Results:**

There was a general perception that QCN had improved health data documentation and use in the learning facilities, achieved through coaching, learning and building from pre-existing initiatives. QCN also enhanced the data elements available by introducing a broader set of quality indicators. However, the perception of poor data quality persisted. Factors negatively affecting data quality included a lack of integration of QCN data within routine health system activities, the perception that QCN was a pilot, plus a lack of inclusive engagement at different levels. Both individual and system capabilities needed to be strengthened.

**Conclusion:**

There is evidence of QCN’s contribution to improving data awareness. But a lack of inclusive engagement of actors, alignment and limited skill for data collection and analysis continued to affect data quality and use. In the absence of new resources, integration of new data activities within existing routine health information systems emerged as the most important potential action for positive change.

## Introduction

Universal health coverage, reflecting access to high quality services for all, is a key milestone of the Sustainable Development Goals (SDGs). However, the quality of care provided to populations in many low-and middle-income settings is sub-optimal; a considerable body of evidence exists to demonstrate that a large burden of maternal, newborn and child deaths can be attributed to poor care [1–3].

A well-functioning health information system is recognized as one of the six building blocks of health systems, which is understood as an interconnected body [4] where delivering high quality health care services requires generating, monitoring and utilisation of quality data as the backbone of health service [1,3,5]. Good quality data was defined to include completeness, timely reporting, accuracy and reliability [1]. In the absence of quality data, poor healthcare decisions and patient outcomes can occur [4].

Responding to the need to improve the quality of maternal and child health (MCH) services alongside the quality of health data, in 2017 WHO and global partners launched a quality improvement initiative ‘The Network for Improving Quality of Care for Maternal, Newborn and Child Health’ (QCN) [6]. The Quality of Care Network sought to reduce in-facility maternal, neonatal and stillbirth case fatality rates by 50% in 5 years initially in nine countries, namely Bangladesh, Cote d’Ivoire, Ethiopia, Ghana, India, Malawi, Nigeria, Tanzania and Uganda. Underlying the network was the assumption that a network of global, national and local network of actors, including facilities, could improve quality through the diffusion of innovation and behavioral change, and generating and sharing quality data [7]. In Ethiopia, the Ministry of Health (MoH) identified interested partners such as Institute for Health Care Improvement (IHI), WHO, Clinton Health Access Initiatives (CHAI), USAID- Transform Primary Health Care (PHC) & Health in Developing Regions (HDR) and created a technical working group, selected 48 learning sites and started implementation in 2018 [6].

As a QCN member, Ethiopia adopted 15 common core QCN indicators for tracking progress in facility-based care for mothers and newborns, categorised to cover provision of care, experience of care and availability of WASH [6]. The strong focus of QCN on tracking data was expected to improve data documentation, sharing and use towards achieving improved quality of care. Data components were collected in the learning facilities and expected to be integrated with the existing health information system such as the Health Management Information System (HMIS) and District Health Information System (DHIS2) [8].

This manuscript focuses on the experience of Ethiopia with a special emphasis on the generation and use of QCN data. Studies have previously reported that the characteristics of good quality data are sub-optimal in Ethiopia [8], that data falsification exists [9,10], and this negatively affects trustworthiness of data and their use in decision making. The relevant studies reviewed, many of them quantitative, and reporting poor quality and low use of data for decision making. The data obtained through HMIS in the country exhibit poor quality due to individual, mainly lack of knowledge, and organisational factors such as lack of training [9,11–20]. An exploration of the factors behind the problem beyond the routine system and its integration with HMIS/District Health Information System (DHIS2) is limited. In this manuscript, we qualitatively explore how QCN contributed to improve data quality, sharing or use, and the factors observed to influence data quality.

## Method

### Study area

Ethiopia is the second most populous country in Africa with a population of over 101 million [5]. Following the country’s federal administrative system, the health sector is organised in a three tier system, namely, tertiary (specialized hospitals designed to serve about more than 3.5 million population), secondary (general hospitals expected to serve around 1 million people), and primary (includes primary hospitals, which are expected to serve about 60,000–100,000 people; health centers serving 15,000–25,000 people and health posts serving 3000–5000 people) [5]. As of 2022, there were about 17,699 health posts, 3,777 health centers, and 367 public hospitals in the country [21].

Data quality is a key component of initiatives to improve quality of healthcare, although its emphasis is a relatively recent phenomenon in Ethiopia. Health service quality was stipulated as a priority agenda in the 2015 Health Sector Transformation Plan (HSTP) [5], which emphasised equitable access to quality health care and the need for quality data in the health system. In 2016, the National Healthcare Quality Strategy (NHQS) [2] was launched to operationalise the quality agenda that paved a way for the establishment of quality structure at federal (Health Services Quality Directorate), regional (case team), district (focal), and facility levels (unit). Important partnerships forged between the MoH and international partners working in healthcare quality such as WHO and Institute for Healthcare Improvement (IHI). The generation and use of quality data were boldly mentioned in the second HSTP (2020/21–2024/25). In both HSTP I and II, information revolution was one of the priorities emphasising the production, availability and use of quality data in the health sector, with a special focus on strengthening HMIS and DHIS2 [10].

The Quality of Care Network (QCN) learning sites in Ethiopia involved 17 hospitals, a 31 health centers in 8 regions and a city administration, namely, Afar, Amhara, Oromia, Gambella, Benishangul Gumuz, Southern Nations Nationalities and Peoples, Tigray, Sidama, and Addis Ababa [22]. Under the overall leadership of the MoH, Quality and the Clinical Service Directorate, the implementing partners distributed the learning site facilities among themselves for provision of technical and financial support.

This analysis was nested within a national MNH Quality of Care Strategic Roadmap implementation, which state ‘supporting quality data system and feedback loops’ as its core objective [8] and a multi-country QCN evaluation study [23], for which data were collected from 41 of the 48 QCN health facilities, excluding those in the Tigray Region, as well as national and subnational implementers. This analysis accessed qualitative data collected from national and regional QCN implementing and supporting partners and from four purposively selected learning facilities located in Oromia, Benishangul Gumuz, Addis Ababa, and Afar regions. These four facilities were selected from the 41 network facilities based on the examination of QCN monthly data (data completeness, facility maternal and neonatal mortality and stillbirths improvement) plus perceived differences in the performance of QCN, a generalised indicators were derived from QCN implementation guideline [6]. In conversation with the MoH, two best and two least performing health centers and hospitals (facilities with optimal/minimal reporting and in which there is high/low decline in maternal, neonatal mortality and still birth) were selected. Considering QCN’s goal of 10% improvement per year, a reduction of greater than 10% in those facilities with good completeness were considered as best and those with high magnitude of increase in mortality has been taken as worst. In addition, an attempt has been made to include cases from both type of facilities (hospital and health center) and from the facilities located around the center and peripheral regions.

### Study design and data sources

Key informant interviews, observations, and document review data were collected in two rounds, during January-March 2021 and September-December 2022, which enhanced the depth of information and helped to investigate the multifaceted factors influencing quality of data at various levels. Initially, the data collection was focused on the general QCN evaluation study, where data quality emerged as a new theme. The second round of data collection sought to explore such data gaps.

#### Key informant interviews (KII)

Key informants with unique and expert knowledge of the issue under investigation due to their participation in the network were purposively selected. To sustain the diversity of views, all QCN members/quality focal persons in the country working at national (this includes, WHO, IHI, CHAI, USAID, Transform Health in Developing Regions (HDR), Transform Primary Health Care Unit (PHCU), UNFPA, UNICEF), all regional health bureaus where QCN was operating, and the Maternal and Child Health (MCH) unit/quality focal person/HMIS officer at the selected health facilities were also included. In view of data saturation, two iterative rounds of fieldwork have been made and a total of 40 KIIs were conducted, 18 at national, 15 at regional, and 7 at facility levels over the two rounds.

#### Facility observations

Two rounds of non-participant observations were carried out in four health facilities to substantiate the data obtained through KII. An observation checklist was prepared that documented the existence and function of essential services, QCN related practices, and data handling and reporting processes. In order to get a complete picture of data process, archive office, facilities registry, QCN data recording and reporting templates have been observed and a discussion has been made with some of the health facility officers.

#### Document review

We also triangulated the data collected through KII and non-participant observation by document reviews. Relevant documents included national level strategies, guidelines, standards, and reports. QCN facilities monthly reports, QCN Learning District Implementation Guide 2018, Ethiopian National Health Care Quality Strategy (2016–2020), Ethiopian Health Sector Transformation Plan I & II, and National MNH Quality of Care Roadmap (2017/18–2019/20) were some of the documents reviewed. In addition, a QCN data set at the MoH has been used to observe data quality and triangulated with the data obtained through KIIs.

The data collection tools have been developed by the study team targeting at understanding the overall QCN implementation and the general QCN data tracking system. The interview guide has been structured along the major themes: the evolution of the network, its relation to the existing health programmes, its contribution to health service quality improvement, and the engagement of stakeholders. The data for this particular paper emerged from the third theme, where the study participants discussed the overall QCN role in improving the quality of health service, which includes data generation and use. The general guiding questions were prepared in English, and then translated into Amharic and Afaan Oromo. The data have been collected, transcribed, translated, and analysed by two of the authors, AAT and ATB. AAT, the lead author of this paper, has PhD in sociology with an extensive experience in qualitative research in public health and social science while ATB was a MPH nutrition and both worked as QCN researchers.

### Data analysis

The qualitative data analysis begun with the work of transcription and translation from local languages to English and theme development. Initially, verbatim transcription was made in the language in which the interview was conducted, predominantly Amharic and Afaan Oromo, followed by translation into English, which enhanced the quality of the data. Moreover, the transcription/translation work has been conducted by the data collectors themselves to maintain its natural setting. The data have been stored in a password protected computer reserved for the QCN project at EPHI. Then a codebook was prepared by the study team using a deductive and inductive approach, guided by the theory of diffusion of innovations [7], the PRISM framework [25], literature review [1–4,8,9,18,20,22] and focusing on addressing the two research questions. The existing frameworks in improving data quality focus on individual and organisational factors. As informed by the PRISM framework, emphasis has been paid on the domains of technical, organisational, behavioural, and data processes during data collection and analysis. We used these domains to understand factors affecting the quality of data. The team then discussed the codebook and a trial coding exercise was undertaken before modifying and finalising. Using this framework, thematic data analysis approach was followed where issues such as data collection tools and their integration to routine health information system, quality of data, medical record, and human resource or infrastructure were considered as health system factors and considered as the major themes. Coding was then conducted by the research team themselves using Nvivo 12, and the results were presented, analysed, and discussed thematically.

#### Ethical considerations

This study was conducted with the permission of the Ethiopian Public Health Institute Institutional Review Board (ref: EPHI-IRB-240-2020) and the London School of Hygiene and Tropical Medicine ethics committee (ref 17541). All interviews and observations were conducted by obtaining a written informed consent. The personal identity of the institutions and respondents were anonymised throughout the data analysis process.

## Results

Network implementation was the main theme in the general QCN study, under which quality of data emerged as a new sub-theme. Network accomplishments with regard to data quality, alignment with the routine health system, QCN data quality, data reporting and use have been the key sub-themes discussed in this section.

### Contribution of QCN towards improved data quality

The importance of data in health care was recognized with the intention to revolutionise data generation and use [5]. MoH analysis suggests that data accuracy, timeliness of reporting and completeness continuous to be a challenge, although data are increasingly used by health officials [2]. This was also reflected by our study participants, with a regional respondent describing that *‘this year [2021] it [data quality]is a flagship initiative in hospital transformation and has got attention but still there is a quality concern’* (Government_subnational_Round 2_04). Hence, QCN was introduced in the country in this context to improve quality of MCH including data handling and reporting.

There was a general perception among the study participants that QCN had improved health data documentation and use in the learning facilities, supporting the emerging policy agenda on better health data quality in Ethiopia. A national government informant specifically noted that the network was perceived to have added value to the skills of individuals in data management and use. Participants mentioned maternal and neonatal mortality tracking and monthly reporting systems that had been initiated by the QCN team at the MoH. Another national government informant substantiated that QCN had improved the tradition of documentation and data use in decision making through training, inter-facility learning, and development of regular reporting formats.

A strength of QCN was to have built on the already existing quality initiatives established by the MoH and other partners such as WHO and IHI. For instance, we observed that one of the best performing health centers posted a paper related to data documentation in the office, which read *‘if it is not documented, it is not done’*. In addition, according to a health facility worker, QCN had worked with the existing quality committees in the learning facilities, comprised of 5–8 people from different departments, who regularly followed-up on overall service provision data.

QCN also introduced new types of information to the data monitoring system. These new data elements helped to provide a more holistic view of health care quality, including documentation of maternal and neonatal mortality by cause, predischarge counseling, interest of pregnant women to have companion during labour/delivery, physical or verbal abuse, newborn birth weight documenting, and the existence of WASH in delivery facilities. But insufficient integration of these new data elements presented a challenge to data quality.

### Insufficient integration of QCN data within the routine health information system

As a member of the global QCN, Ethiopia had adopted and implemented 15 common core quality indicators developed by WHO [6], which assumed to improve the quality of maternal and newborn care. These common core indicators were collected manually by health care providers in parallel with HMIS and DHIS2, not integrated with the routine health information system. A national government informant described that *‘we are using a parallel reporting system; QCN data is not part of the DHIS-2; only very few indicators come in the routine health information system’* (Government_National_Round 2_08). As a result, providers engaged in collection and reporting of the common core indicators in addition to the routine data, and this was perceived as a burden. Variables related to WASH and experience of care (including mortality by cause, counseling, and companion during labour/delivery, and abuse) were not integrated with the DHIS2. The perceived reasons behind the lack of integration include a lack of leadership commitment and a lack of shared responsibility for QCN. Some national respondents considered QCN as a pilot that needed political approval before inclusion in their routine activities. There were also conflict of interest between units in the health system over ownership of the programme (Quality Directorate Office was perceived as owner while MCH and Regional Health Bureaus perceived as excluded). A national level respondents described that ‘*there is a gap in working these two programmes (MCH and Quality directorate) together and there is a problem in collaboration and how the programme should sustain*.’ (Government_National_Round 2).

According to a respondent from the MoH, the low level of engagement of some key units at national and subnational/regional levels negatively influenced the integration of QCN data in the routine health information system. Some regional informants mentioned lack of transparency over data sharing. Further, there was evidence of mistrust between MoH and Regional Health Bureaus over the use of budget for QCN related activities, including improved data systems. The Regional Health Bureaus perceived themselves to be excluded from QCN decision making. A national level government key informant said, ‘*due to bureaucracy, we decided to manage learning sessions by our own instead of giving them money. Initially, we send budgets for regions but the money may not reach there timely or may repurposed for other purpose. For instance, in 2018, we have sent budget, but the money sent was repurposed at regional level. We realized this inefficiency and we haven’t sent them the money then … the budget sent for coaching were also repurposed for emergency issues. So, regions have financial autonomy, once they offered, they have the right to use it autonomously and they may repurpose*’ (Government-national-Round2–07).

There was also a low level of awareness about the future direction of QCN, with some government actors describing it as just a pilot test. He illustrated as, ‘*We are not taking QCN seriously since piloting is not completed [until early 2022]. We overlooked it since this is a pilot stage…, there is a problem of engagement and linkage. It could be due to low level of awareness. The network doesn’t have influence since we have a perception that it is at a pilot stage, so we simply wait for the outcomes of the pilot rather than to learn from it*.’ (Government_national_Round2_08).

Data sharing was also key to QCN global, national and local learning. The QCN data collected from facilities was reported up in the system via email, being prepared in excel sheets provided by the MoH. It was also observed that some facilities used telegram to send reports and receive feedback from top officials. Feedback was planned back from MoH to facilities to reflect completeness, timely reporting, and trustworthiness of some figures included in the report. This system, which included data visualisations, helped the MoH to regularly monitor trends in maternal, newborn and stillbirth mortality in the learning facilities.

However, facility and regional level respondents mentioned that feedback and comments from the national level were either irregular or not provided at all. Moreover, data management related trainings and coaching were limited due to lack of budget from MoH or withdrawal of NGOs supporting QCN. As a result, some facilities stopped collecting and reporting QCN data. There was limited knowledge of analysing and using the data for collective learning, and respondents mentioned a lack of capacity or required skill at the local level for using data for decision making.

But when you go to facility there are challenges regarding the network, how to analyze the data and use it. It is very challenging; you train them but still the problem is there. This is a problem across the country. Due to this the data issue has got a due attention and a focus in the second strategy. (Government_National_Round2_07)

Within Ethiopia, the experience of data sharing between network members and other facilities were minimal. A couple of national QCN partners described a lack of trust and recognition by the MoH to expand/upgrade its scale to other facilities.

### Factors perceived to influence data quality

Despite issues around integration, QCN was observed to have contributed to increased awareness of data documentation and reporting. Nonetheless, there continued to be concerns about data quality. The study participants mentioned the whole processes of data, which start from the general archive system of the facilities as the main influencing factor of data quality. Some providers interviewed mentioned that they kept maternal files in their office considering the main archive center is not digitalized and hardly retrieved. The study respondents reported that the staff doing the recording had little knowledge, skill, or resources. One health facility worker elaborated that there was a shortage of skilled manpower and noted that usually the people assigned to this position were non-professionals with low education level and mostly not familiar in use of computers. Data activities were generally thought to be *‘the neglected unit and lack due attention’* according to a health facility worker.

The organisation of data records was perceived to be poor. A health facility worker mentioned that *‘if you want to retrieve 4 or 5 years data, it is very difficult to get’* (Local_Round2_04). We further observed in one of the least performing facilities long delays when retrieving files from the archive department for mothers who were admitted to the maternity ward *‘it took 20–25 minutes to bring the files. The reasons include there being a large number of patients (both pregnant and other patients with no separate filing/archive for mothers); and a shortage of staff working in the archive’* (Facility observation_facility_3_ Round2). One of the midwives in the facility described that *‘there was a time when the files may disappear’* due to poor file management. Moreover, due to a lack of appropriate facilities such as internet, computers, and electricity, data were said to not be recorded or could be lost or delayed in sharing. It was also observed that some of the paper based registry books were too old and some of the pages were lost. An informant in a facility mentioned that *‘there is only one computer in the facility. MCH staff do not have computer and internet access and this limits their ability to record and share the data’* (Facility_Round2_Interview 03).

The respondents also mentioned cases of data unreliability. There were instances of over or non-recording and reporting. A subnational key informant expressed his suspicion over the reliability of data reported to his office: *‘Usually inflated data are being reported to our office. If you actually go to field and check, there is a difference between the report and actual thing. There are many complaints in the community’* (Government_subnational_Round2_04).

It was noted that some providers *‘cook’* data reports if they had been unable to conduct required data recording activities. QCN leaders track the learning facilities performance mainly based on timeliness, completeness, and level of mortality decline. The facility leaders were aware of this criteria and try to present a positive image, and as a result, some facility leaders manipulate data.

The data collected are usually inflated. For instance, they usually don’t ask the service satisfaction of a delivered woman; it is either blank or filled latter with an assumption. They report it as if the community is satisfied. So we don’t trust all data coming from all facilities and would usually lack reliability.(Government_subnational_Round2_04)

Sometimes works accomplished but not recorded, other times unaccomplished works were falsely documented. A facility informant described that there was a time when maternal mortalities were reported while there was no mortality in reality, and there was also a time when the actual maternal mortality in the facility was not reported.

The commitment and trust of the documenter or reporter was thought to play a role, with some informants describing a lack of transparency in some learning facilities. Multiple other possible reasons were put forward by participants, related to data entry errors due to lack of basic knowledge or training, negligence of the officer, or fear of their supervisors. Differences were also thought to be due to leadership, commitment, and human or financial resource capacity.

Differences in data completeness were observed between regions, types of facilities, and components of the indicators. Monthly reports of 41 QCN learning facilities illustrated that the total number of facilities with at least three missing values for the data reported in 2019 were higher for Oromia region (6 out of 10 facilities) and SNNP (4 out of 7 facilities) and also were higher for hospitals than health centers. Regarding maternal mortality reports, the missing values was high in Oromia (52) followed by SNNP (41), Amhara (35), and Afar (26) and least in Addis Ababa (0). Furthermore, indicators related to WASH and experience of care were usually incomplete, since these indicators required separate assessments to be made. A facility informant elaborated this in detail as: *‘The interview part of the common core indicators is challenging; in that we are filling by our own assessment. The interview part requires somewhat counseling; we have problems with that. Due to the existing workload, our team is also responsible for OPD, and since there is a shortage, on duty time we cannot do. So, some data are being collected and some not’* (Government_Facility_Round2_02).

## Discussion

This study intended to explore how QCN facilitated data quality improvement in MCH services in Ethiopia and factors influencing the quality of data. The study found that QCN was perceived to improve data quality, sharing, and use through coaching, learning, and sharing between the QCN implementing facilities. It also introduced new data elements to MCH service which aimed to improve quality of maternal and newborn care, which include WASH, experience of care comprising client-providers interaction. However, these data were not integrated to the existing system due to low awareness and lack of inclusive engagement among health system actors and due to the assumption that QCN was only a pilot.

Our study found a variety of individual and health system factors affecting QCN data quality. At the individual level, the staff technical capacity to understand data collection tools, and low levels of on-the-job training for data handling and analysis highly influenced the quality of the data. As a result, some QCN data points exhibited incompleteness. A perception of *‘cooking’* data was reported, negatively affecting trust for data sharing and use. There were differences with regard to data quality between regions, types of facilities and by the common core indicators. High missing values were observed in Oromia, SNNP, Afar, and Amhara for the maternal mortality indicators. Indicators related to WASH and experience of care was perceived to be high for all regions, being influenced by lack of commitment in collecting the data. And these differences were attributed to a lack of resources or leadership or the active support of partners.

In congruent with the existing frameworks [25], both micro- and macro-dimensions have been highlighted in the previous Ethiopian studies [9,18–20]. Although the previous studies including MoH and WHO [3,4] have emphasized issues around completeness, timeliness and accuracy or reliability as features of data quality, our study explored the factors behind these challenges and the interplay of micro and macro issues, emphasizing the systems supporting data documentation processes playing a key role. At the macro level, the overall policy context including the existence of decentralized structures supporting data quality works, recognition of the QCN and coaching and technical/financial support have been the key factors. It has been revealed that insufficient attention and prominence has been given to data archives and supporting infrastructure in addition to the capacity of care providers. At the micro-level, factors around understanding, capability, commitment, and trust have been prominent. Many of the previous studies conducted in the country exclusively quantified quality of the routine health information system [9,10,12,14,15,17–20,24,25]. This particular study presented the issue of data integration as a key factor of data quality. It was found that data generated from non-routine health interventions such as QCN were not integrated with the routine system due to lack of awareness and knowledge. The study also recognised the role of key network actors in the MoH system structure as influencing factors of quality data. Most of the QCN learning facilities were supported by international and national partners by facilitating or funding learning sessions, coaching, and supportive supervisions, which contributed to data handling, completing and timely reporting. However, in some learning facilities most of the interventions were not sustainable due to the withdrawal of supporting partners and the limited engagement of regional, zonal and district health offices, although the MoH had an intention to scale up the programme.

Strength and weakness

The data for this study was collected from QCN learning facilities and QCN stakeholders at national, sub-national and local levels. The two iterative rounds of data collection enhanced the depth and trustworthiness of the data. However, we did not include people from health facilities which were not members of QCN and did not compare the quality of data over time. This study presents findings only from providers’ perspective.

## Conclusion

This study presents evidence of QCN contributing to data quality through inter-institutional learning, sharing, and building on existing activities. QCN introduced new data elements in the Ethiopian health system which was thought to add value to quality MCH care endeavors. However, the engagement of multiple actors at the regional, zonal, and district levels was minimal, and QCN data were not integrated within the routine health information system.

Actors were concerned that poor data quality persisted under QCN, with insufficient training taking place in a context of mainly paper-based records and outdated archive systems. Most of the limiting factors presented were both micro (individual capabilities) and macro (system) issues, which include health system support such as attention to archive units, training, provision of data handling facilities, and shortage of budget. Although the issue of the quality data generation got attention, QCN was perceived to be owned by a single unit with limited awareness and engagement of related actors in the health system and this has attributed to low responsibility and commitment of officials and providers. In the absence of new resources, integration of new data activities within routine systems emerged as the most important potential action for positive change.

## Context of the paper

This paper is part of the main **QCN Evaluation** of the WHO- Network for Improving Quality of Care for Maternal, Newborn and Child Health. The overall research project tried to address: How does a multi-country, multilateral network focused on specific health care improvements evolve and what shapes its ability to achieve its goals? This evaluation study took place in Bangladesh, Ethiopia, Malawi and Uganda from 2019–2022. This manuscript is the country specific, focusing on the role of the network in improving the data quality and its influencing factors in Ethiopia.
